# Comparison of mitotic cell death by chromosome fragmentation to premature chromosome condensation

**DOI:** 10.1186/1755-8166-3-20

**Published:** 2010-10-19

**Authors:** Joshua B Stevens, Batoul Y Abdallah, Sarah M Regan, Guo Liu, Steven W Bremer, Christine J Ye, Henry H Heng

**Affiliations:** 1 Center for Molecular Medicine and Genetics, Wayne State University School of Medicine, Detroit, USA; 2 Karmanos Cancer Institute, Detroit, USA; 3 Department of Pathology, Wayne State University School of Medicine, Detroit, USA

## Abstract

Mitotic cell death is an important form of cell death, particularly in cancer. Chromosome fragmentation is a major form of mitotic cell death which is identifiable during common cytogenetic analysis by its unique phenotype of progressively degraded chromosomes. This morphology however, can appear similar to the morphology of premature chromosome condensation (PCC) and thus, PCC has been at times confused with chromosome fragmentation. In this analysis the phenomena of chromosome fragmentation and PCC are reviewed and their similarities and differences are discussed in order to facilitate differentiation of the similar morphologies. Furthermore, chromosome pulverization, which has been used almost synonymously with PCC, is re-examined. Interestingly, many past reports of chromosome pulverization are identified here as chromosome fragmentation and not PCC. These reports describe broad ranging mechanisms of pulverization induction and agree with recent evidence showing chromosome fragmentation is a cellular response to stress. Finally, biological aspects of chromosome fragmentation are discussed, including its application as one form of non-clonal chromosome aberration (NCCA), the driving force of cancer evolution.

## Introduction

Morphological characterization of abnormal chromosomes represents an important aspect of medical genetics. Modern medical genetics was firmly established following the successful identification of trisomy 21 in Down syndrome [1]. Since then other types of chromosome aberrations have been identified and linked to various diseases including cancer [2]. Diverse abnormal chromosomal structures have been detected including; translocations, duplications, deletions, inversions, fusion, double minute chromosomes, chromosomal breaks, sister chromatid exchange (SCE), apoptotic bodies, and defective mitotic figures [3,4], but shared chromosomal aberrations have received the most attention [5,6]. Identification of recurrent chromosomal markers in patients has greatly contributed to both diagnosis and patient management, particularly in certain types of blood cancers with dominant recurrent changes [7].

Despite decades of intensive effort, identification of commonly shared chromosome aberrations for most diseases has been difficult [4-9]. In contrast, large numbers of nonrecurring chromosome aberrations have been observed but generally ignored based on the viewpoint that these non-recurrent chromosomal aberrations are insignificant genetic noise [5,6,9-11]. Increasing evidence demonstrates that non-recurrent genetic change including non clonal chromosome aberrations (NCCAs), are the main contributing factor in diseases involving system instability and specifically have been identified as the driving force of cancer progression [5,6,8-11]. According to the recently established genome theory, stochastic chromosome alteration plays a key role in both organismal and in somatic cell evolution. Stochastic genome alterations have also been closely associated with a large number of diseases [12]. Thus, systematic characterization of various types of NCCAs and their application to clinical diagnosis is of importance.

Chromosome fragmentation is one type of recently identified NCCA that requires more attention [13]. Chromosome fragmentation is a non-apoptotic form of mitotic cell death, and is observed from an array of cell lines and patient tissues. Its occurrence is associated with various drug treatment or pathological conditions. New data also illustrates that it is a programmed cell death as PARP is readily degraded during the process of chromosome fragmentation, where chromosomal fragmentation occurs as a general response to cellular stress including oxygen stress, ER stress and various pathological stresses (Stevens et al submitted).

The identification of chromosome fragmentation as a form of cell death is new to the field, but the morphological description of chromosome fragmentation is not. With similar morphologies, chromosome pulverization and PCC have been extensively studied since the 1960s [14,15]. Many studies have confused the identity of pulverization and PCC due to their similar morphologies. In regards to reassessing the classification of chromosomal abnormalities, the further analysis of chromosome fragmentation, a form of mitotic cell death, has renewed interest in phenomena such as chromosome shattering and pulverization. The identification of chromosome fragmentation as a distinct form of cell death shows that the issue of PCC/pulverization/shattering also needs to be reexamined to establish the relationship among different cytogenetic identities. In this review these often confused processes are analyzed from both a morphological and mechanistic aspect. Finally, the relationship between chromosome fragmentation and genome system dynamics is discussed in light of the genome theory.

### Brief over views of chromosome fragmentation and PCC

#### Chromosome fragmentation

**Figure 1:**
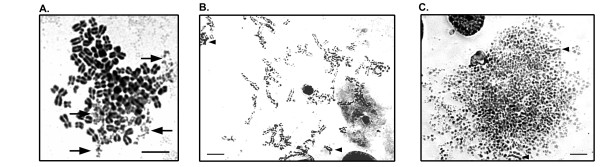
**Examples of the various stages of chromosome fragmentation in a subclone of HCT116 human colon carcinoma cells**. **A**. Early stage chromosome fragmentation where most chromosomes are intact. Chromosomes being degraded are denoted by arrows. The degree of condensation occurring in the chromosomes should be noted and are very condensed when compared to the small degree of condensation occurring in the examples of S-phase PCC in figure 2. **B**. Mid-stage chromosome fragmentation where most chromosomes still display some chromosomal morphology despite multiple breaks. Nearly complete chromosomes are also identifiable (arrowheads). **C**. Late stage chromosome fragmentation where most chromosome morphology has been lost due to degradation despite the fragments still retaining a high degree of condensation. A small number of chromosomes are nearly intact (arrowheads).

Chromosome fragmentation is a major form of mitotic cell death that is identified through abnormal cytogenetic figures [4,13]. These figures contain chromosomes with multiple breaks, similar to the morphology of S-phase PCC which is discussed below. During the process of chromosome fragmentation the number of chromosomal breaks increases until all chromosomes are completely degraded. They often show lighter density Giemsa or DAPI staining than normal chromosomes stained in parallel indicating the loss of chromosomal material. Morphologically, chromosome fragmentation can be grouped into at least three groups: early fragmentation where few chromosomes are broken; mid-stage fragmentation where a significant number of the chromosomes have been fragmented; and late stage fragmentation where all or most of the chromosomes have been fragmented which suggests it is a progressive process (Figure 1). This progressivity is illustrated by time course experiments where more late stage chromosome fragmentation is detectable with longer treatment [13].

Chromosome fragmentation is non-apoptotic as evidenced by differences in morphology and other biochemical differences [13]. Apoptosis results in cytogenetic figures typified by small round clusters of spontaneously condensed (not mitotic condensation) fragmented DNA [16]. Chromosome fragmentation is further distinguished from apoptosis as it is not reliant on caspase activity, nor is it affected by Bcl-2 over-expression [13]. Mechanistically it occurs as a general response to stress (Stevens et al. submitted). The stresses that have been shown to induce chromosome fragmentation are diverse in nature ranging from commonly used chemotherapeutics with various molecular mechanisms, to the targeting of specific stress pathways such as the unfolded protein response, to changes in culture conditions including temperature change and oxygen concentration, to genomic instability, and to various pathologies including cancer. The link between chromosome fragmentation and genomic instability is significant as the cellular system is forced to change in response to genome change, creating stress within the cell. Chromosome fragmentation like other forms of cell death, functions as a response to said stress in order to maintain genome integrity.

#### Premature Chromosome Condensation (PCC)

PCC was first described in 1970 by Johnson and Rao, using cellular fusion methods developed previously [14,17]. PCC was shown to occur when two asynchronous cells fuse if one of these cells was in mitosis. This was originally carried out using the UV inactivated Sendai virus [14]. Methods were subsequently developed to induce fusion using chemicals such as polyethylene glycol [18]. More recent mitotic catastrophe work has utilized cell lines transfected with the HIV envelope protein and the CD4 receptor to induce fusion [19]. Aside from fusion based PCC, a second method of induction has also been reported where chemical inhibitors of the cyclin b/cdk1 inhibitor, protein phosphotase C have been utilized, negating the need for cellular fusion [15]. Inhibitors of protein phosphotase C include okadaic acid and calyculin A [15,20,21]. Recent reports have demonstrated the utility of calyculin A in cytogenetic analysis, particularly within tumors [15]. Tumors are collections of asynchronously dividing cells, often with a low mitotic index rendering cytogenetic analysis difficult. Calyculin A allows for cytogenetic analysis of tumor cells without culture as all G2/M cells can be analyzed.

PCC results in three distinct chromosomal morphologies determined by the cell cycle progression of the induced cell [22,23]. G2 PCC chromosomes are similar in morphology to normal mitotic chromosomes with sister chromatid attachment and no breaks or gaps, though chromatids are longer than metaphase chromosomes, similar to chromosomes in early prophase. G1 PCC is similar in appearance to G2 PCC, except there are no sister chromatids as replication has not been started. In S-phase PCC chromosomes appear as condensed pieces of chromatin or chromosomes with multiple breaks and gaps, depending on how far replication has proceeded [23]. The main focus of this review will focus on S-phase PCC, as its morphology is often confused with that of chromosome fragmentation.

Fusion and chemical inhibitor based methods of inducing PCC are similar in molecular mechanism and morphology. Both methods result in exposure of an interphase cell to the activated cyclin b/cdk1 complex (also known as mitosis promoting factor or MPF) [15]. In fusion PCC the activated complex is provided by the mitotic cell, whereas in inhibitor induced PCC, the complex is prematurely activated through inhibition of protein phosphatases that dephosphorylate cyclin b in order to keep it inactive [15,24]. This difference results in two small differences in morphology. The first is that in fusion based PCC nuclei/chromosomes from the fused cells are always in close proximity to each other [14,25]. The second difference between the two is that G1 PCC rarely occurs in chemical inhibitor based PCC as little or no cyclin b can be found in the cell until mainly S-phase [15]. In the case of fusion PCC, G1 PCC is readily detectable as the mitotic cell provides enough activated cyclin b/cdk1 complex to drive condensation of unreplicated G1 chromosomes.

PCC has applications in the research lab and the clinic. Chemical induction of PCC can be used to increase the number of mitotic cells within tumor samples, allowing for rapid karyotyping with only very short term culture being required [15]. PCC can also be used in order to perform cytogenetic evaluation of quiescent and senescent cells. This will likely gain in importance as the measure of karyotypic diversity is used on a more regular basis in cancer treatment and monitoring [26]. In addition, PCC has been used in making clinical predictions [27]. Elevated PCC can also be detected in various disease conditions including cancer, microcephaly and mental retardation [28,29]. Chromosome fragmentation has been often confused with PCC, and we have recently illustrated that chromosome fragments are frequently observed in various types of experimental and pathological stresses including various diseases. Therefore, an examination of whether these previously observed PCCs are in fact chromosome fragmentation is now needed. One possibility is that the process of inducing PCCs may also induce chromosome fragmentation and PCCs may also be an indicator of system stress. Clearly, further investigation is needed to address these issues.

#### Chromosome pulverization/shattering

Multiple reports analyze two phenomena called chromosome pulverization or chromosome shattering [30-32]. These terms have been used synonymously with PCC or in some cases have been associated with cell death. A variety of mechanisms have been used to induce pulverization, frequently including viral infections or drug treatments. A summary of these reports is found in Table 1. Taken together, these reports all show highly varied mechanisms of induction of pulverization, and few offer clear-cut evidence that the given conditions actually induce PCC. When considered in light of chromosome fragmentation, this confusion can be resolved. The vastly ranging mechanisms of chromosome pulverization and/or shattering are all diverse stresses impacting the cellular system. When that system is unable to adapt to or recover from the stress, the cell must then be eliminated. This is carried out through a number of death processes including chromosome fragmentation (Stevens et al, submitted). Thus chromosome pulverization often has little to do with PCC but rather is chromosome fragmentation.

**Table 1:** Various stresses that have been linked to chromosome pulverization/shattering

Factors of pulverization/shattering	Species	**Ref**.
Shattering due to UV light and caffeine exposure	Chinese Hamster	[44]
Maintenance of diploid karyotype in PA-1 cells by removal of tetra ploid cells	Human	[62]
UV exposure	*Tradescantia paludosa*	[66]
Cells with herpes-like virus given doses of colcemid	Human	[67]
Exposed to various doses of tritiated thymidine	Chinese Hamster	[68]
Exposure of male mice to methyl methanesulfonate previous to fertilization of female mice. Shattering seen in filial cells	Mouse	[69]
Infection of lymphocytes with JM-V herpes-virus	Chicken	[70]
Treatment with fungicide N-trichloromethylthio-phthalimide	Human	[71]
Pulverization due to UV light and caffeine	Chinese Hamster	[72]
Exposure to alpha-amanitin	Rat	[73]
Doxorubicin treatment. Pulverization inhibited in drug resistant cells.	Human	[74]
Herpes simplex virus type 1 infection	Human	[75]
Hepatitis B infection. Pulverization occurs in both a hepatocellular cell line derived from a tumor and in peripheral lymphocytes from the patient.	Human	[76]
Herpes simplex virus type 1 infection. Endoreduplication was noted. Also HSV infection increased the mitotic index.	Human	[77]
Friend leukemia cells exposed to high levels of adriamycin.	Mouse	[78]
Following incubation of cells with heat labile DNA polymerase A in S phase at 39°C which were then cultured in a permissive temperature.	Mouse	[79]
Photo-irradiation of G2 or early prophase cells.	CHO	[80]
Hepatitis B integration and genomic instability	Human	[81]
N-methyl-N'-nitro-N-nitrosoguanidine (MNNG), sodium selenite and caffiene treatment of CHO cells	Chinese Hamster	[82]
Culture of a fibroblast line generated from a patient with xeroderma	Human	[83]
Associated with ubiquitin-activating enzyme E1 activity	Mouse	[84]
Vaccination against hog cholera virus	Pig	[85]
Radiaton exposure in bone marrow. Treatment with WR-2721 and/or Ocimum sanctum extract reduced the amount of pulverization.	Mouse	[86]
2-methoxyestradiol, an endogenous metabolite of estrogen	Human	[87]
Following vaccination of pigs for swine fever	Pig	[88]
Streptozotocin treatment	Human	[89]
Viral infection in pigs	Pig	[90]
Vitamin C treatment of lymphocytes	Human	[91]

Previous reports have shown chromosome fragmentation but have called it chromosome pulverization/shattering, therefore one option is to continue to use the term(s) shattering/pulverization, however there are a number reasons not to do so. They include the following: First, descriptions of pulverization have focused largely on identification of later stage fragmentation events. The term chromosome fragmentation encompasses all stages of this mitotic cell death while pulverization has only been used to describe late stages. Second, DNA fragmentation is used to describe the genome degradation that takes place during apoptosis [29]. Therefore, in keeping with the term fragmentation to denote cell death related genome breakdown, the term chromosome fragmentation is used. The term chromosome fragmentation has been used synonymously with pulverization in limited reports, thus there is a historical basis for the use of the term chromosome fragmentation [33]. Finally, chromosome pulverization has been used synonymously with PCC to the extent that they have been inextricably linked. Thus using the term chromosome fragmentation averts any erroneous links.

### Similarities and differences between chromosome fragmentation and PCC

Chromosome fragmentation and PCC can be confusingly similar, so what exactly differentiates these phenomena? In the following discussion, chromosome fragmentation and PCC will be differentiated first by morphology and then by mechanism (Table 2).

**Table 2:** Identifiable differences of chromosome fragmentation and PCC

Chromosome fragmentation	PCC
**Morphologic**	
Single cell involvement	If fusion induced, normal, intact mitotic cells will be in close proximity to fragmented cells
Can affect single chromosomes	Impacts entire genome regularly, except in limited multinucleated cells
Results in chromosome degradation	Unknown, may activate chromosome breakdown
Chromosome morphology lost as process progresses	Chromosome morphology dependant on position in cell cycle
Differential cut size	Differential condensation states
**Mechanistic**	
Occurs during mitosis	Occurs in interphase cells exposed to active MPF
Not inhibited by roscovitine	Inhibited by roscovitine
Induced by stress during mitosis	Induced by cell fusion or activation of MPF
γ-H2AX positive	γ-H2AX negative
No active DNA incorporation	Actively incorporating DNA

#### Morphology

S-phase PCC and chromosome fragmentation each produce striking mitotic figures where gaps/breaks are interspersed among pieces of condensed chromosomes [13,14,34]. Subtleties that differ between each can be useful in distinguishing chromosome fragmentation from PCC. An easy way to identify fusion based PCC is through close association of a normal mitotic cell with the cell undergoing S-phase PCC. Well-controlled cytogenetic preparation with restrained hypotonic treatment used to make chromosome spreads often does not result in a broken membrane, so cells that have fused membranes will retain their close association when cytogenetic slides are prepared. Careful slide preparation, where samples are diluted enough so that resulting cell suspension drops do not land too close to one another, but not so much so that there is insufficient material to analyze, is key to ensuring chromosome fragmentation is not considered PCC. Although fusion based PCC is the easiest form of PCC to distinguish from chromosome fragmentation, chemical induced PCC can also be distinguished based on morphology.

Most mitotic figures from cells undergoing chromosome fragmentation show variable degrees of fragmentation across the chromosomes where chromosomes that are completely degraded are found within figures that also contain completely intact chromosomes and every degree of degradation between these extremes also can be seen (Figure 1). In human cells, PCC figures have less variability as replication takes place relatively similarly across the entire complement of chromosomes. There are, however, limited examples of single chromosome pulverization in highly heterochromatinized chromosomes that undergo delayed replication [35]. Also, multinucleated cells can undergo asynchronous replication leading to PCC in a limited number of chromosomes and this is discussed in the following paragraph [36]. In cases of early stage chromosome fragmentation there may be only one or a handful of chromosomes that are fragmented. This does not typically occur during PCC as the progression of replication is relatively consistent across all chromosomes. Chromosome fragmentation is often induced in the presence of microtubule dynamics inhibitors such as colcemid or docetaxel. This treatment can result in overly condensed chromosomes, and chromosome fragmentation is often detectable in spreads with highly condensed chromosomes. Chromosomes that are prematurely condensed do not over-condense and in fact are typically under condensed. This distinction offers yet one more way to discriminate chromosome fragmentation and PCC. Furthermore, electron microscopy of PCC shows that the areas in S-phase PCC figures that appear to be gaps are not actually broken, but rather are single strand DNA that has not yet been replicated [37]. Strand breaks are detectable in the case of chromosome fragmentation by γ-H2AX staining and not in the case of PCC [13]. We thus anticipate that electron microscopy of chromosome fragmentation will show strand breaks between fragments.

Multinucleated and micronucleated cells are an interesting case where chromosome fragmentation and PCC can occur and affect a limited amount of the genome within the multinucleated cell. Multinucleated cells are a common occurrence in tumors, indicative of genomic instability, not commonly shared and are therefore one type of NCCA [38,39]. In multinucleated cells, although each nuclei is located within the same cell, each nuclei can replicate at different times [36]. The differential replication can result in entrance into mitosis in one nuclei while another may still be in S-phase. Mitotic entry of one nuclei causes exposure of the other nuclei to MPF driving it into premature mitosis [36]. This occurs in under 50% of multinucleated cells with apparent pulverization, thus it is more common for multinucleated cells to eliminate extraneous nuclei through chromosome fragmentation than for that material to undergo PCC [36]. One method of determining whether a micronucleus is undergoing PCC is through detection of DNA incorporation, morphological assessment of cells can also indicate multinucleate associated PCC may be inducing single chromosome fragmentation. First, for single or few cell chromosome fragmentation to be PCC cells must be multinucleated and multinucleated cells should be apparent within the observed population. Second, within the chromosome spread it is unusual for a pulverized micronucleus to be surrounded by intact chromosomes. If a fragmented chromosome is completely surrounded by intact chromosomes the fragmented chromosome is unlikely to be derived from a different nucleus undergoing PCC. Lastly, chromosome fragmentation and PCC are distinguishable by γ-H2AX staining. If the pulverized chromosome(s) in question is not positive for γ-H2AX, it is likely PCC, whereas if it is, it is being degraded by chromosome fragmentation.

It should be pointed out that chromosome fragmentation is a unique phenomenon of mitotic cell death, and should not be confused with just any type of chromosomal breakage. Chromosome fragmentation involves chromosome breaks, but this does not mean they are the same. For example, the recently reported phenomenon that there appears to be chromatin tethers that hold broken chromosomes together is likely not associated with chromosome fragmentation [40]. It would be difficult to imagine that the massive fragmentation that occurs during chromosome fragmentation could be rescued by this tether mechanism, as studies have clearly linked chromosome fragmentation to mitotic cell death [13]. It is likely that only a very limited number of breaks could be overcome in a few generations by this tether mechanism.

#### Mechanism

**Figure 2:**
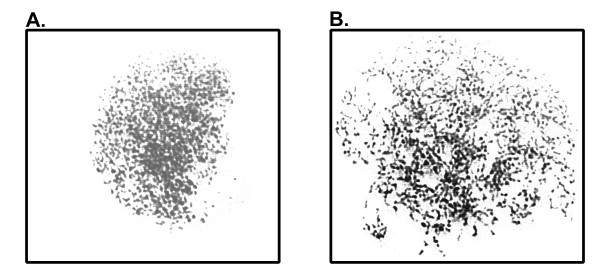
**Examples of S-phase PCC induced by 80 nM calyculin A treatment**. Later S-phase PCC (**A.) **and earlier S-phase PCC **(B.) **are shown. In later S-phase PCC chromosomes are nearly completely replicated and have fewer gaps than early S-phase PCC. In both stages chromosomes are not highly condensed.

Further work has also separated the two distinct phenomena. Chromosome fragmentation was not inducible in double thymidine blocked S-phase cells that remained in interphase (Stevens unpublished observation). This indicates that these cells arrested properly in either S-phase and/or G2 and were not able to prematurely enter mitosis. PCC, when the morphology is similar to chromosome fragmentation, occurs specifically in cells in S-phase [15]. Interspersed segments of replicated and unreplicated DNA manifest as condensed DNA with intervening empty spaces similar in morphology to chromosome fragmentation (Figure 2). If chromosome fragmentation occurs in mitosis, there should be no DNA incorporation, whereas if it is related to S-phase DNA replication it should be detectable. DNA synthesis can be directly monitored by the inclusion of the thymidine analog, bromo-deoxyuridine (BrdUrd). In mitotic arrested cells induced to undergo chromosome fragmentation no BrdUrd incorporation was shown, however BrdUrd was detectable in asynchronous cells induced to undergo PCC by calyculin A [13]. This suggests that, despite the morphological similarity between chromosome fragmentation and PCC, they are in fact distinct processes.

PCC results in the abnormal activation of MPF, so inhibition of this activation would inhibit PCC. Roscovitine is a purine analog that functions as an inhibitor of the G2 to M transition through inhibition of CDK1 activity [41,42]. Roscovitine treatment should inhibit PCC, but not any mitotic processes, as cells that are already in mitosis are not inhibited by treatment. Mitotic cells treated with roscovitine and 1 ug/ml doxorubicin concurrently while still under colcemid pressure readily undergo chromosome fragmentation (Stevens et al. Submitted). This further supports the conclusion that chromosome fragmentation and premature chromosome condensation are not the same process. Thus chromosome fragmentation and S-phase PCC can be distinguished based on both morphological and mechanistic assessment.

### Chromosome fragmentation, PCC and the G2 checkpoint

Though chromosome fragmentation and PCC can typically be differentiated based on morphological and mechanistic differences there is potential overlap of the two. This potential overlap occurs at the G2 checkpoint where any potential DNA damage is repaired prior to mitosis. Induction of PCC occurs through the abnormal activity of MPF subsequently subverting the G2 checkpoint. Traditionally, caffeine has been used to inhibit the G2 checkpoint and concurrent treatment with various drugs or radiation can result in chromosome fragmentation [30,43-45]. UCN-01, is a recently developed chemotherapeutic that reduces the ability of cells to arrest in G2 [46]. It has shown great promise in *in vitro *studies and is now in active clinical trials [47-49]. Concurrent treatment with UCN-01 and other drugs such as topoisomerase inhibitors potentiates cell death to a synergistic level [50]. Chromosome fragmentation is induced strongly in doxorubicin and UCN-01 treated cells. On one hand, these cells are prematurely entering mitosis in that they should arrest at the G2 checkpoint, but because the checkpoint is inhibited these damaged cells enter mitosis. On the other hand, these cells have completed S-phase and do not contain the large blocks of un-replicated DNA that typifies S-phase PCC cells. Differentiation of the two phenomena comes from PCC resulting from the artificial activation of MPF. In this case MPF is activated at the normal time; however inhibitory signals of the G2 checkpoint are unable to slow the process. Cells then enter mitosis with irreparable damage and are eliminated through the degradative process of chromosome fragmentation. Similarly, the same process can happen when cells lacking G2 checkpoint activity have replication inhibited by treatment with aphidicolin [51]. In this system ATR null cells are unable to arrest following incomplete replication due to aphidicolin treatment leading to premature entry into mitosis of cells with under-replicated DNA. Again, the key to differentiating chromosome fragmentation and PCC here is that following mitotic entry, chromosomes undergo degradation. It is not known if this degradation occurs in the cellular fusion or chemical induced forms of PCC. Further work is needed to determine if this happens or not.

### Chromosome fragmentation and mitotic catastrophe

Mitotic catastrophe is often used to refer to mitotic related cell deaths. However there is no distinct definition of this type of cell death. Reports vary widely and include deaths that occur directly during mitosis, or occur following a failed mitosis [52-56]. Due to the ambiguity of the term mitotic catastrophe, its use has been cautioned against [57], but because of the pervasiveness of mitotic catastrophe within the literature and its potential connections to chromosome fragmentation and PCC, it will be briefly discussed.

Certain models of mitotic catastrophe, particularly those that are based on cell fusion, are in fact suggestive of PCC. However others are based on loose G2 checkpoints [50,51]. When HCT116 cells are treated with UCN-01 and doxorubicin, chromosome fragmentation is detectable. This suggests that cells entering mitosis with high enough levels of damage undergo chromosome fragmentation. Cells with damage below this level may undergo mitotic catastrophe and die in the subsequent G1 phase through apoptotic mechanisms. However, under normal conditions these cells should arrest and not enter mitosis, and are in a sense undergoing PCC. This PCC is different from classical S-phase PCC in that this occurs after the DNA has been replicated in S-phase, and in that it is not driven through forced MPF exposure, either through fusion with mitotic cells or activation caused by chemical inhibition. In fact, in HCT116 cells treated with UCN-01, but without doxorubicin, no increase in chromosome fragmentation is detectable even though it is likely that some cells enter mitosis through a G2 block over-ride. Early studies describe what was believed to be S-phase PCC following viral infection [58]. It was assumed that this phenomenon was the same as S-phase PCC induced through cellular fusion because of the similarities in morphology. It was hypothesized that this phenomenon was a response by the infected cells to eliminate the viral infection through self destruction [34]. It is possible that some of the observations were actually of chromosome fragmentation, which was induced because of genome change and/or stress induced from the viral load, and that these cells were responding in a protective manner.

### Chromosome fragmentation is a non-clonal chromosome aberration

Following the discussion of the importance of chromosome fragmentation in cell death, it is necessary to mention its importance in genome research as well as future diagnostic use. Importantly chromosome fragmentation is one manifestation of the previously ignored non-clonal chromosome aberrations. NCCAs reflect the level of genomic instability within a population of cells, which according to the principles of the genome theory, are the driving force of cancer evolution and drug resistance [4-6,8-12,59,60]. Chromosome fragmentation has been shown to occur in response to genomic instability serving to eliminate that instability and to protect the cellular bio-system. System instability can be induced through a number of cellular stresses, including single gene change. It is important to note that association between chromosome fragmentation and specific gene change is often context dependant. In the case of p53, p53 function itself was shown not to affect the induction of chromosome fragmentation with doxorubicin [61]. In another case however, when the functional status of p53 is changed, spontaneous levels of chromosome fragmentation also change. When the status of p53 is changed from non-functional to functional, there is a transient die off of cells through both chromosome fragmentation and apoptosis as stability is attained within that system [13]. This is what happens in the H1299v138 system where cells with a temperature sensitive p53 mutation grow well at the restrictive temperature where p53 does not function. Upon temperature shift and re-establishment of p53 function, cells die via chromosome fragmentation and apoptosis until the population of cells has adapted to the new system that incorporates p53 function [13]. In the MAD041 *in vitro *model of spontaneous transformation, p53 function is abrogated in the germ line [12]. During transformation in this model, stochastic chromosome instability increases until cells reach crisis and a dominant clone is selected between passage 20 and 30. Throughout this progression, chromosome fragmentation increases and decreases in accordance with genomic instability measured by non-clonal chromosome aberrations. Taken together, this data indicates that chromosome fragmentation reflects system dynamics as well as playing an important role in eliminating unstable cells in general.

Increased chromosome fragmentation has also been shown to occur in the PA-1 cell line where chromosome fragmentation takes place in order to eliminate polyploidy cells [62]. Chromosome fragmentation can also influence system dynamics not just by eliminating cells, but also by altering the genomes of surviving cells. This happens in two ways. First one or a small number of chromosomes can be selectively degraded during the chromosome fragmentation process leading to changes in chromosome number. More drastic change can also occur when the process does not complete. Incomplete chromosome fragmentation can result in reattachment of chromosomal fragments resulting in the creation of very complex chromosomes with multiple translocations also known as karyotypic chaos. Apoptotic cell death can also lead to system change through horizontal transfer, as DNA from apoptotic cells is taken up and incorporated by neighboring cells [63]. Whether chromosome fragmentation can result in horizontal transfer remains unknown. Further work is needed to address this issue. What is clear however, is that chromosome fragmentation can not only change cellular systems by elimination of selected cells, but chromosome fragmentation can also result in changed karyotypes. The karyotype defines the cellular system in both somatic cell evolution and organismal evolution [64,65]. Therefore, chromosome fragmentation can influence the creation of new cellular systems which can lead to cancer and other diseases.

Although chromosome fragmentation is important to maintain system stability, there are many redundant systems within cells that ensure that cells maintain homeostasis and are not permanently damaged at the genome level. Chromosome fragmentation becomes activated when cells reach mitosis with extensive damage or in the face of extensive stress. Chromosome fragmentation works in conjunction with other regulators of cellular integrity, such as the cell cycle checkpoints and other forms of programmed cell death (apoptosis and autophagy), to maintain the cellular system identity. Therefore, chromosome fragmentation can be applied as a clinically useful measure of NCCAs. Furthermore, chromosome fragmentation frequencies have also been shown to roughly approximate total NCCA levels. This is significant, as chromosome fragmentation analysis can be used as a relatively quick and inexpensive substitute for spectral karyotyping in patient monitoring. Although chromosome fragmentation indices may not hold all the information that SKY analysis could reveal, it does give an overall estimate of the genomic instability contained within a given sample. This information combined with information from the same slides such as mitotic index, frequency of apoptotic cells, and frequency of micronuclei may be useful in determining treatment modalities in many diseases including cancer.

## Conclusion

Chromosome fragmentation is a form of mitotic cell death that was only recently characterized. In some manifestations, chromosome fragmentation can appear quite similar to PCC. However in depth analysis shows that chromosome fragmentation and PCC are in fact quite different phenomena, though at times they may be linked to each other. They are distinguishable by morphological and mechanistic analyses. Distinguishing between chromosome fragmentation and PCCs is significant. Demonstration that they are two independent biological phenomena means that chromosome fragmentation can now be used as an easily applied cytogenetic index to measure mitotic cell death that could have clinical and basic research applications. In addition, despite decades of extensive studies, there is little known about the biological significance of chromosome pulverization. By separating chromosome pulverization from PCC, a long standing puzzle can now be solved. It now seems that the biological meaning of PCCs is rather limited. Well controlled experiments are needed to further separate PCC and chromosome fragmentation. In addition, chromosome fragmentation has been identified as a form of NCCA, which is the driving force of cancer progression, and therefore analyses of chromosome fragmentation in clinical settings could lead to better treatments and outcomes in cancer therapy.

## Abbreviations

BrdUrd: Bromo-deoxyuridine; MPF: Mitosis promoting factor; NCCA: Non-clonal chromosome aberration; PCC: Premature chromosome condensation; SCE: Sister chromatid exchange; SKY: Spectral karyotyping

## Competing interests

The authors declare that they have no competing interests.

## Authors' contributions

JBS and HHH wrote the paper. SMR contributed table 1. BYA, SMR, GL, SWB, CJY edited the paper and contributed expanded ideas. All authors have read and approved the final manuscript.
